# Efficient Identification of Causal Mutations through Sequencing of Bulked F_*2*_ from Two Allelic Bloomless Mutants of *Sorghum bicolor*

**DOI:** 10.3389/fpls.2017.02267

**Published:** 2018-01-12

**Authors:** Yinping Jiao, Gloria Burow, Nicholas Gladman, Veronica Acosta-Martinez, Junping Chen, John Burke, Doreen Ware, Zhanguo Xin

**Affiliations:** ^1^Cropping Systems Research Laboratory, Agricultural Research Service (USDA), Lubbock, TX, United States; ^2^Cold Spring Harbor Laboratory, Cold Spring Harbor, NY, United States; ^3^USDA-ARS NAA Plant, Soil and Nutrition Laboratory Research Unit, Cornell University, Ithaca, NY, United States

**Keywords:** *Sorghum bicolor*, mutation mapping, bloomless, epicuticular wax, cutin synthase

## Abstract

Sorghum (*Sorghum bicolor* Moench, L.) plant accumulates copious layers of epi-cuticular wax (EW) on its aerial surfaces, to a greater extent than most other crops. EW provides a vapor barrier that reduces water loss, and is therefore considered to be a major determinant of sorghum's drought tolerance. However, little is known about the genes responsible for wax accumulation in sorghum. We isolated two allelic mutants, *bloomless40-1* (*bm40-1*) and *bm40-2*, from a mutant library constructed from ethyl methane sulfonate (EMS) treated seeds of an inbred, BTx623. Both mutants were nearly devoid of the EW layer. Each *bm* mutant was crossed to the un-mutated BTx623 to generated F_2_ populations that segregated for the *bm* phenotype. Genomic DNA from 20 *bm* F_2_ plants from each population was bulked for whole genome sequencing. A single gene, Sobic.001G228100, encoding a GDSL-like lipase/acylhydrolase, had unique homozygous mutations in each bulked F_2_ population. Mutant *bm40-1* harbored a missense mutation in the gene, whereas *bm40-2* had a splice donor site mutation. Our findings thus provide strong evidence that mutation in this GDSL-like lipase gene causes the *bm* phenotype, and further demonstrate that this approach of sequencing two independent allelic mutant populations is an efficient method for identifying causal mutations. Combined with allelic mutants, MutMap provides powerful method to identify all causal genes for the large collection of *bm* mutants in sorghum, which will provide insight into how sorghum plants accumulate such abundant EW on their aerial surface. This knowledge may facilitate the development of tools for engineering drought-tolerant crops with reduced water loss.

## Introduction

Forward genetics is a powerful approach to identifying gene mutations that cause phenotypes of interest, and ultimately elucidating the biochemical and molecular mechanisms and signaling processes underlying plant development and adaptation. The forward genetic approach begins with physical or chemical mutagenesis of a pure (inbred) line, followed by screening for mutants with the desired phenotype. The resultant mutants are outcrossed to another pure line with extensive DNA polymorphism relative to the mutagenized parental line. Linkage of the mutant phenotype with DNA markers is analyzed, using as many markers as possible, until the mutation is mapped to a very small region flanked by two markers. The success of this process, also referred to as map-based cloning, depends on two critical factors: a large segregating mapping population and dense DNA markers with the resolution to delimit the mutation into a small region. Several hundreds to thousands of accurately phenotyped F_2_ plants must be analyzed with large number of DNA markers in order to narrow the mutation to a region harboring only a few genes (Jander et al., 2002). The entire region is then sequenced in order to identify the gene that carries the causal mutation. To confirm the identity of the gene, the wild-type gene is introduced into the mutant through transformation to determine whether it can complement the mutant phenotype. This last step can be bypassed if two or more independent mutant alleles are identified that carry unique mutations in the same gene because the odd of finding two independent mutations by chance on the same gene that lead to the same phenotype is near zero (Jander et al., 2002).

Next-generation sequencing (NGS) techniques provide massive high-resolution genotypic data very quickly (Metzker, 2010), and can thus replace the costly and time-consuming linkage analysis required by conventional map-based cloning (Schneeberger and Weigel, 2011). Two general approaches have been developed for using NGS to identify causal mutations. The first strategy is represented by two similar approaches, mapping-by-sequencing (ShoreMap) (Schneeberger et al., 2009; Hartwig et al., 2012) and next-generation mapping (NGM) (Austin et al., 2011). These approaches are very similar to conventional map-based cloning. First, a mapping population is generated by outcrossing the mutant to a divergent line with sufficient DNA polymorphism, and homozygous mutants are selected from the segregating F_2_ population. Then, equal quantities of genomic DNA from each of the selected lines are pooled for NGS analysis, according to the principle of bulk segregant analysis (BSA) (Michelmore et al., 1991). Because only homozygous mutants selected from the mapping F_2_ population are subjected to whole-genome sequencing, the single-nucleotide polymorphic (SNP) markers surrounding the causal mutation will be derived predominantly from the mutant parent. Consequently, the region containing the causal mutation will form a “valley” devoid of polymorphic markers from the divergent parent. The region containing the causal mutation can be narrowed further with sophisticated bioinformatics tools, sometimes, to a single gene (Austin et al., 2011; Schneeberger and Weigel, 2011).

The second approach is represented by isogenic mapping-by-sequencing (MutMap), a variation of ShoreMap, designed to map mutant phenotypes subject to modification by genetic background (Abe et al., 2012; Hartwig et al., 2012; Zhu et al., 2012). In MutMap, the mutant is crossed to a wild-type plant of the same genetic background as the mutant, rather than to a divergent line as in ShoreMap, to avoid interference from a divergent genetic background. Homozygous mutants are selected from the backcrossed F_2_ population and bulked for whole-genome sequencing. After identification of SNPs, the SNP ratio (i.e., the proportion of all short reads that contain the mutated SNPs) is plotted along each chromosome. The causal mutation for the phenotype is expected to have a SNP ratio of 1 (i.e., 100% mutant), whereas unlinked background mutations are expected to have ratios around 0.5. For mutations close to the causal mutation, the SNP ratio should vary from <0.5 to 1 depending on genetic distance. This approach is particularly useful for mapping induced mutations (Abe et al., 2012; Zhu et al., 2012). Because major mutations capable of causing phenotypes are rare in induced mutant populations (Greene et al., 2003; Henry et al., 2014; Jiao et al., 2016; Krasileva et al., 2017), it is often possible to narrow down the candidates to one or a few mutations using 20–50 homozygous mutants selected from a segregating F_2_ population (Abe et al., 2012; Takagi et al., 2015).

Sorghum is the fifth most important crop in the world, providing food, feed, and forage to humans and agricultural animals in many areas around the world (http://www.fao.org/home/en/). In addition, due to its excellent tolerance of drought and high-temperature stress, high water use efficiency, and high productivity, it has become an increasingly important crop for bioenergy production (Rooney et al., 2007; Vermerris, 2011; de Siqueira Ferreira et al., 2013; Mullet et al., 2014). Sorghum accumulates higher levels of epicuticular wax (EW) on its aerial surface than most other crops, and this trait is considered to make an important contribution to sorghum's high water use efficiency and drought tolerance (Jordan et al., 1984; Jenks et al., 1992; Premachandra et al., 1994; Samdur et al., 2003; Burow et al., 2008; Uttam et al., 2017). Many sorghum mutants that are devoid of EW, named “*bloomless*” (*bm*), have been isolated over the years but few genes responsible for the deposit of massive EW on its aerial surface have been identified (Weibel, 1986a,b; Peters et al., 2009; Jiao et al., 2016).

Here, we report an efficient method for identifying causal gene mutations by whole genome re-sequencing of bulked F_2_ populations, and demonstrate the approach using two independent alleles of sorghum *bm* mutants devoid of EW. We chose the *bm* mutants for allelic mutant mapping because we have isolated a large collection of *bm* mutants from the sorghum mutant library generated with ethyl methane sulfonate (EMS) in an elite inbred line BTx623 and the characteristic shining green sheath that can be easily distinguished from the sheath of wild type sorghum (Burow et al., 2008, 2009; Peters et al., 2009). Our method offers a tool to identify most *Bm* genes in sorghum. These *Bm* genes may facilitate improvement of water use efficiency and drought tolerance, not only in sorghum, but also in other crops (Weibel, 1986a,b; Peters et al., 2009; Jiao et al., 2016; Uttam et al., 2017).

## Materials and methods

### Generation of sorghum pedigreed mutant library and selection of *bm* mutants

The sorghum pedigreed mutant library was established as described (Xin et al., 2008). During the development of the mutant library, each head row at the M_3_ stage was evaluated for mutants that were devoid of EW on the sheath of the flag leaf at the boot stage. The *bm* mutants could be easily identified from the shiny boot when the flag leaf was fully expanded. Over the last few years, over 100 independent *bm* mutants have been identified from this mutant population (Jiao et al., 2016). The putative *bm* mutants were crossed to BTx623_ms8_, a near-isogenic line of BTx623 with a nuclear male sterile mutation (Xin et al., 2017). The nature of the mutation (recessive/dominant) was determined in the F_1_ plants from the cross: if the F_1_ plants produced high levels of EW, the mutation would be recessive, whereas if the F1 plants had the *bm* phenotype, the mutation would be dominant. All *bm* mutants isolated so far are recessive. Only *bm* mutants that exhibited a single Mendelian segregation ratio (1 *bm*: 3 WT) in F_2_ progeny were considered to be confirmed *bm* mutants. Efforts to determine the allelic groups of these *bm* mutants are ongoing, but are far from complete due to the large number of complementation crosses required.

### Phenotype analysis

EW coverage was analyzed by scanning electron microscopy (SEM) at the College of Arts & Sciences Microscopy of Texas Tech University. Fresh leaf and sheath tissues were mounted on a sample holder and imaged directly at 15 KeV accelerating voltage under air pressure of 30–90 Pa on a Hitachi S-4300 field emission environmental scanning electron microscope. To determine the wax load, BTx623 and two *bm* mutants were planted in a greenhouse at 28/20°C (day/night) temperatures on May 25, 2017. At boot stage, when the flag leaf was fully expanded, the plants were photographed and subjected to EW analysis. EW from the leaf and sheath of the second leaf from the top (the leaf right below the flag leaf) was extracted by dipping a cut sample of ~6 cm^2^ in 6 ml of hexane for 60 s. Samples were dried under nitrogen, subjected to esterification using the MIDI protocol (http://www.midi-inc.com), dried again under nitrogen, and reconstituted in hexane containing an internal standard. Wax analysis was performed using a gas chromatograph coupled with a flame ionization detector (FID).

### Allelic bulked segregant analysis

Two allelic *bm* mutants were selected for this study. Previously, 39 complementation groups of sorghum *bm* mutant have been reported (Weibel, 1986a,b; Peters et al., 2009; Uttam et al., 2017). Therefore, we tentatively named the two allelic *bm* mutants as *bm40-1* and *bm40-2* because their allelic relationship to previously reported *bm* mutants has not been tested. Two F_2_ populations were developed by crossing each *bm* mutant to BTx623_ms8_, a nuclear male sterile near-isogenic line (NIL) of BTx623 (Xin et al., 2017). From the F_2_ populations, 20 *bm* mutants were selected for preparation of genomic DNA according to the method as described in Xin and Chen (2012). Equal amounts of DNA were bulked and subjected to 150-bp paired-end sequencing on an Illumina X-10 instrument (https://en.novogene.com). About 10 Gb of high-quality sequence, corresponding to ~15× coverage of the whole genome, was obtained for each bulked F_2_ population. The data from the two F_2_ population were first analyzed separately using our highly efficient pipeline, ems_mutation, in the Cyverse Discovery App (Merchant et al., 2016) (https://pods.iplantcollaborative.org/wiki/display/TUT/EMS+mutant+sites+identification+Workflow). Briefly, the reads were first aligned to sorghum reference genome version 2 using Bowtie2 in “very-sensitive” mode. Samtools was used to sort and change the format of the alignment file (Li et al., 2009; Paterson et al., 2009). PCR duplications in the reads were masked using Picard-tools-1.113 (http://broadinstitute.github.io/picard/). Variation call process was performed by Samtools and Bcftools (Li, 2011). Only EMS-induced type GC
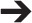
AT single-nucleotide changes supported by 5–100 reads were retained for downstream filtering. Background variations from the parental line were filtered according to two datasets: the variations called from whole-genome sequencing of the parental line BTx623, and the high-frequency (≥0.05) allele variations in the sequenced mutants from the same population (Jiao et al., 2016). Based on the high accuracy of phenotyping, the SNP ratio ([number of reads supporting the mutation allele]/[number of all reads mapped to the loci]) cutoff was set to 1. SNPs with large effects on genes (missense, nonsense, splice_site_acceptor, splice_site_donor) predicted by SNPeff (Cingolani et al., 2012) were retained as candidate causal mutations. The candidate mutations from two allelic F_2_ populations were then compared to determine the overlap.

To analyze the phylogenetic relationship of GDSL lipases, phylogenetic tree was constructed using Mega 7 (Kumar et al., 2016) with ClustalW alignment and the maximum likelihood method from all sequences and orthologs extracted by Gramene BioMart (Tello-Ruiz et al., 2016). Multiple alignments from ClustalW were visualized with MSAviewer (Yachdav et al., 2016).

## Results

### Deficiency of EW in *bm40* mutants

**Figure 1:**
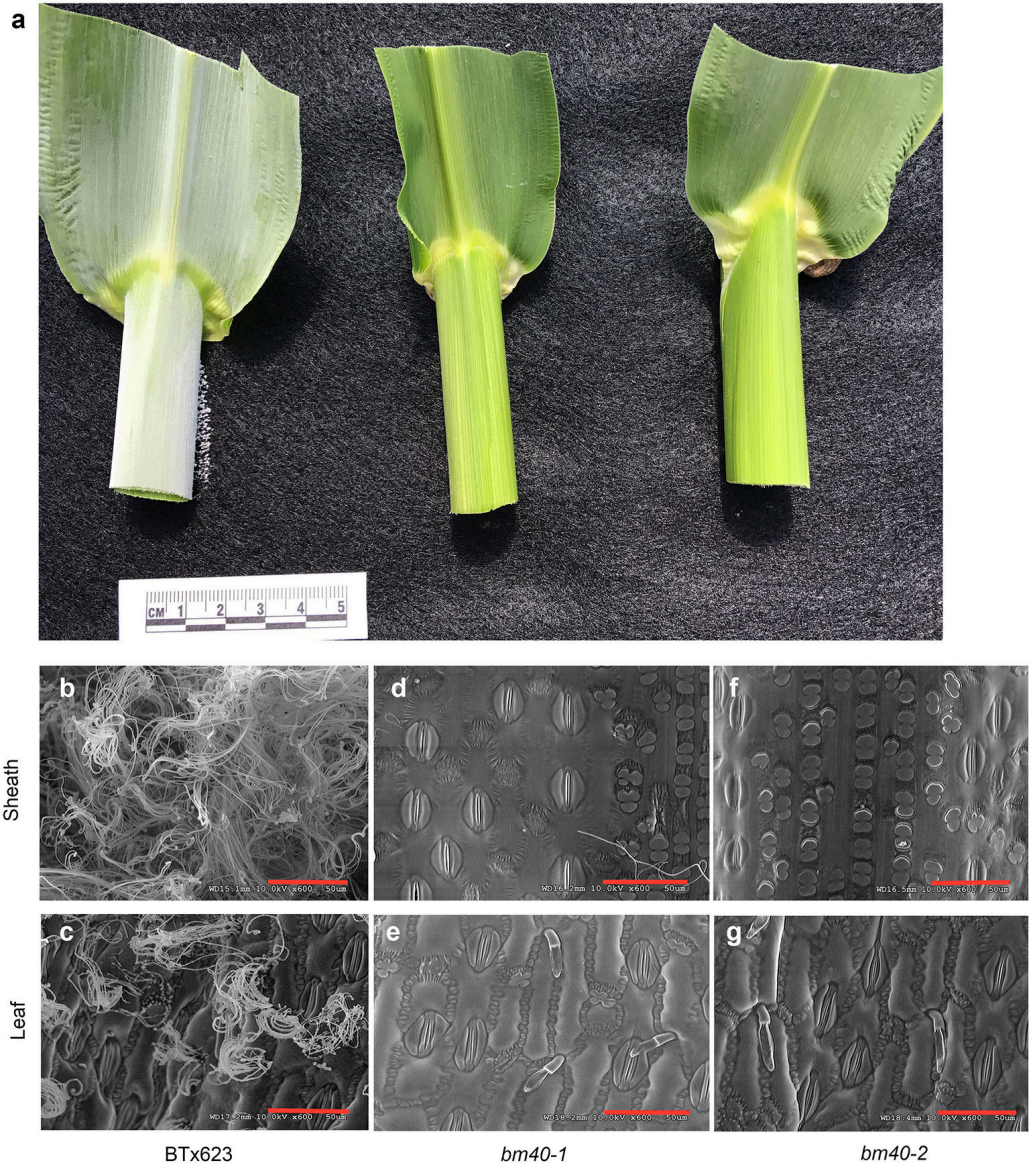
Characterization of the *bm* phenotype. The second leaf from the top was sampled from BTx623 (left), *bm40-1* (middle), and *bm40-2* (right) at boot stage when the flag leaf was fully expanded. The top panel shows photographs of leaf and sheath **(a)**. The abaxial leaf surface and sheath of WT BTx623 are covered with EW, whereas in *bm40-1* and *bm40-2* these organs were almost free of wax. The middle panel shows SEM of sheaths **(b,d,f)**. The bottom panel shows SEM of abaxial surfaces of leaves **(c,e,g)**. Both surfaces were covered with fibrous wax crystals in WT BTx623 but lacked wax crystals in *bm40* mutants.

Two recessive allelic *bm* mutants, *bm40-1* and *bm40-2*, isolated from the sorghum pedigreed mutant library (Xin et al., 2008; Jiao et al., 2016) were used to identify the causal gene for *bm40* mutants. The aerial surfaces of plants of the inbred line BTx623, which was used to generate the pedigreed mutant population, were covered with conspicuous white crystals of EW, especially on the surface of the leaf sheath (Figure 1). In BTx623, the sheath obviously accumulated more EW than leaves (Figures 1a–c). SEM of the WT sheath revealed that the stomata on the sheath were completely covered with fibrous wax crystals, whereas the abaxial surface of leaf blade was only partially covered (Figures 1b,c). In the *bm40-1* and *bm40-2* mutants, the surfaces of both leaves and sheaths were devoid of wax crystals, and the stomata were completely exposed (Figures 1a,d,e,f,g).

To measure wax load in BTx623 and the *bm* mutants, pieces of sheaths and leaves, ~6 cm^2^, were dipped quickly to hexane to extract wax. The extracted EW was quantified by weight after hexane evaporated. In comparison with the wild type BTx623, the wax loads of leaves and sheath surfaces in the mutant were reduced by 80–84% and >92%, respectively (Table 1).

**Table 1:** Analysis of leaf and sheath epicuticular wax (EW) content of WT and bloomless sorghum mutant plants at boot stage, and estimation of the amount of EW reduction in mutants vs. WT.

**Genotype**	**Leaf**		**Sheath**	
	**EW (mg/cm^2^)**	%EW reduction^*^	**EW (mg/cm^2^)**	%EW reduction^*^
WT	2.12 (0.15)	0	5.67 (0.33)	0
*bm40-1*	0.34 (0.15)	84.00	0.44 (0.19)	92.16
*bm40-2*	0.42 (0.15)	80.00	0.44 (0.19)	92.16

*Values inside the parentheses are standard deviation*.

* *%EW reduction was estimated as: = [{WT (EW)- Bloomless1(EW)}/WT (EW)] × 100*.

**Figure 2:**
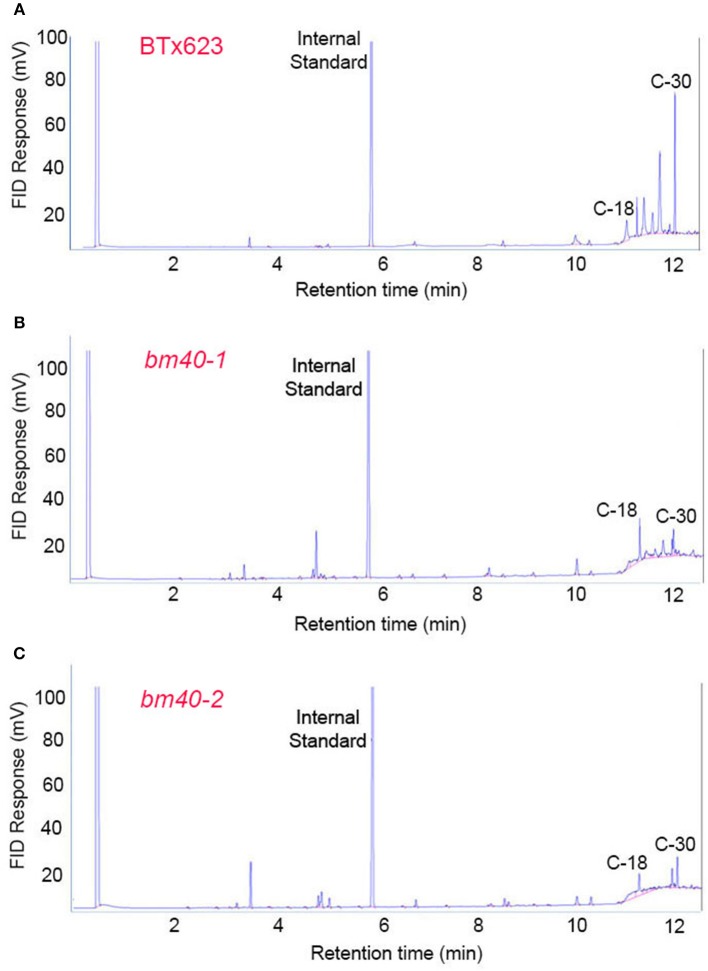
Fatty acid profile of surface wax from wild-type BTx623 **(A)** and the two bloomless mutants **(B,C)**. Epicuticular waxes were extracted by quick immersion in hexane. After esterification, the fatty acids were analyzed by GC (with a FID detector). The levels of long-chain fatty acids (C-30) were greatly reduced in both *bm* mutants in comparison with BTx623.

To examine the fatty acid composition of the extracted EW, the lipids extracted from the surfaces of the WT and *bm* mutants were esterified and analyzed by GC. Consistent with the wax load analysis, BTx623 accumulated large quantity of C30 and other long chain fatty acids (Figure 2A) on the surface of leaf sheath, which were greatly reduced in the *bm* mutants (Figures 2B,C).

### The causal gene in *bm40* encodes a cutin synthase

**Figure 3:**
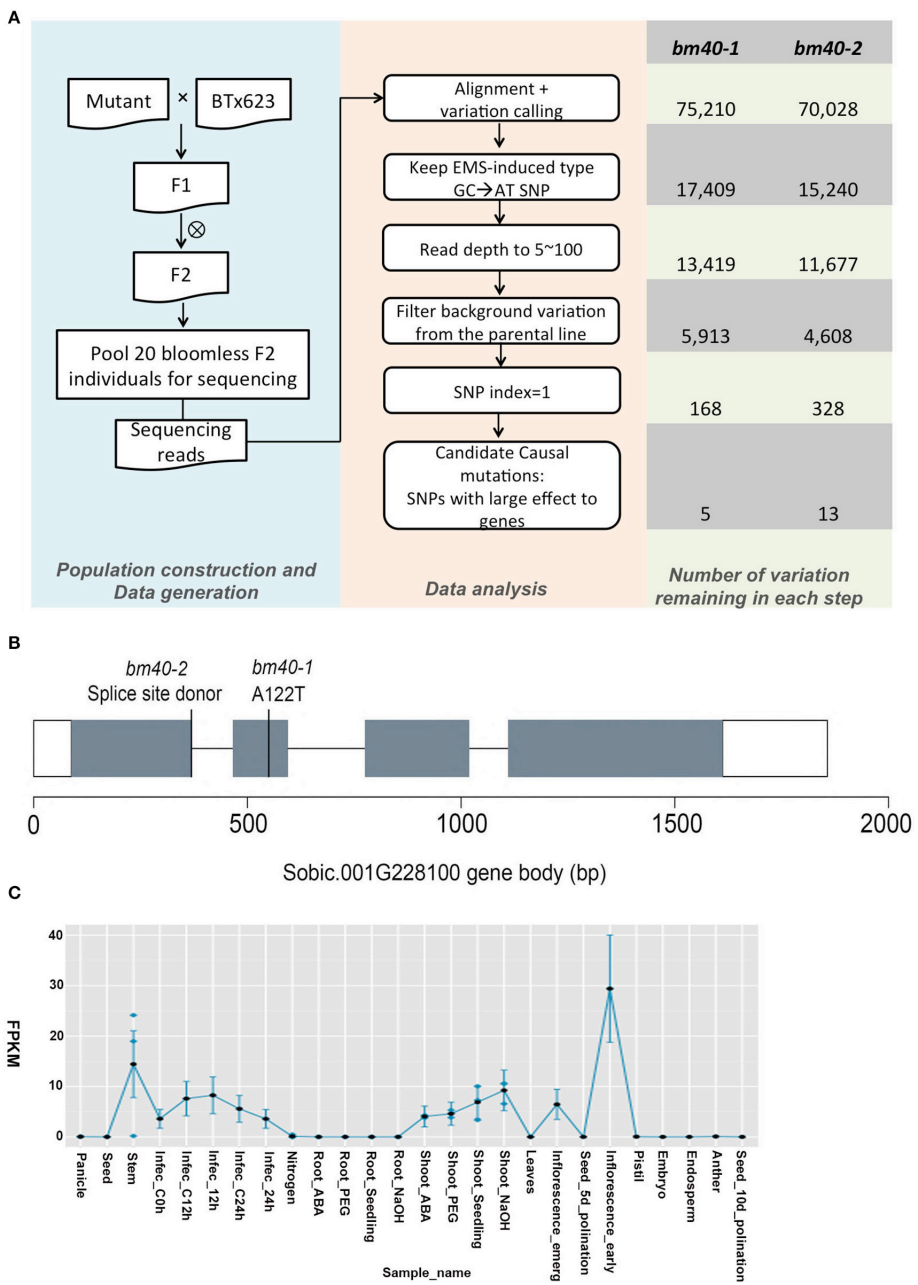
*Bm40* encodes a GDSL-like lipase. **(A)** Schematic overview of bulk segregant analysis and numbers of candidates at each step in the *bm40-1* and *bm40-2* F2 progeny. **(B)** Candidate gene *Bm40*. White and gray boxes represent UTRs and exons. The GDSL domain (amino acid: 44-354) was defined by Pfam (ID: PF00657). The *bm40-1* and *bm40-2* mutations were a missense amino acid change (A122T) and an alteration in the splice site donor of the first intron. **(C)** Expression pattern of *Bm40* from MOROKOSHI sorghum Transcriptome Database: http://sorghum.riken.jp/morokoshi/Data/Sobic.001G228100.html.

To identify the causal mutation, both mutant strains were subjected to BSA (Figure 3A). For each F_2_ population, a total of 20 F_2_ *bm* type individuals were pooled for sequencing, yielding 12 and 18 Gb 150-bp paired-end data for *bm40-1* and *bm40-2*, respectively. After background mutations were filtered out using our high-efficiency data analysis pipeline, 5 and 13 homozygous mutations remained for bulked F_2_ of *bm40-1* and *bm40-2*, respectively. Comparison of the two bulked F_2_ populations revealed that only one gene, Sobic.001G228100, was present in both populations. In *bm40-1*, a G
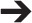
A mutation at genomic DNA position chr01_21842959 results in substitution of alanine at amino acid position 122 with threonine (A122T), whereas in *bm40-2*, a G
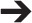
A mutation at position chr01_21843140 alters a splice site after the first exon (Figure 3B).

The *bm40* gene encodes a GDSL-like lipase/acylhydrolase with 383 amino acids, and both mutations are in the GDSL-like lipase domain (Pfam ID: PF00657). The protein is similar to cutin synthase 1 (CD1) in tomato (Yeats et al., 2014). According to the public sorghum expression atlas data (Makita et al., 2015), the gene is highly expressed in aerial tissues but undetectable in roots and seeds, consistent with its role in cutin synthesis on aerial surfaces (Figure 3C).

**Figure 4:**
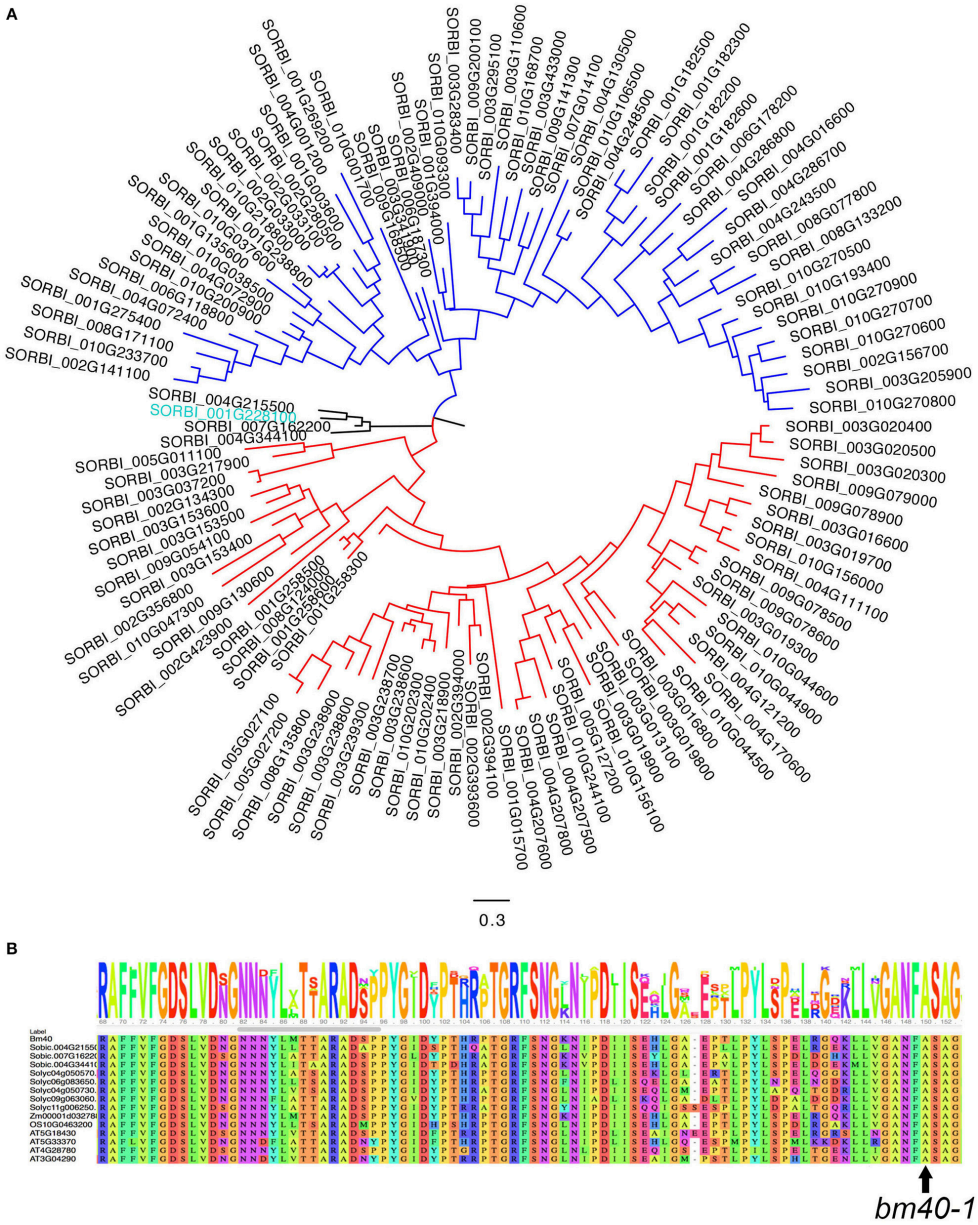
Phylogenetic analysis of *Bm40* gene. **(A)** Phylogenetic tree of 118 GDSL lipases/acylhydrolases from the sorghum genome. Bm40 (blue) is a member of a small clade with four members. **(B)** Alanine 122, mutated in *bm40-1*, is conserved in all four genes in this clade and in the tomato, *Arabidopsis*, sorghum, and rice orthologs.

Querying the sorghum genome with the Pfam domain of GDSL returned a total of 118 genes that could be grouped into three clades (Figure 4A); *Bm40* belongs to a small clade with four members. Alignment of these four proteins with the *Arabidopsis* and tomato orthologs of BM40 revealed that the site of the missense mutation in *bm40-1*, A122T, is conserved across the sorghum paralogs in this clade and *Bm40* orthologs across species (Figure 4B).

The GDSL lipase/hydrolases are a large newly discovered gene superfamily present in many kingdoms of life, including plants (Ling, 2008; Chepyshko et al., 2012; Lai et al., 2017). These enzymes have a broad substrate spectrum and may be involved in a wide range of metabolic pathways. Although large numbers of this family are present in plants, few genes have been assigned specific functions. One member of the superfamily was shown to be the gene encoding cutin synthase, *CD1*, in tomato (Yeats et al., 2012b). *CD1*, which is expressed between the cell wall and cuticle layer, is responsible for synthesis of the polyester component of the cuticular network. *Arabidopsis Cus2*, a homolog of the tomato CD1, is involved in synthesis and maintenance of cuticle ridges on flower petals. Sorghum *Bm40* is closely related to both genes. Moreover, the large reduction in EW in *bm40* mutants was also similar to the tomato *cd1* lines lack of EW (Yeats et al., 2012a).

## Discussion

Here, we demonstrated that sequencing of two independent allelic mutants represents a powerful approach to identifying causal gene mutations in sorghum. Our approach is very similar to MutMap through bulked segregant analysis, described in rice (Abe et al., 2012). Conceptually, MutMap is a straightforward way to identify the causal homozygous mutation in bulked F_2_ plants selected for a phenotype of interest. Because the mutant phenotype is preselected, the causal mutation is expected to present in all short sequence reads covering the mutation site, whereas the ratio of mutant to WT SNPs will be 0.5 for all unlinked mutations. For linked mutations, this ratio varies from 1 to <0.5 depending the distance to the causal mutation. In practice, however, the situation is more complicated. Sequencing errors in the reference genome and pre-existing SNPs in BTx623 prior to mutagenesis will all be identified as homozygous mutations (Figure 3A), making it challenging to determine which homozygous mutations are causal. In a previous study, we sequenced BTx623 and 256 selected lines from a mutant population generated in this line (Jiao et al., 2016). From sequenced BTx623 and mutant lines, we annotated over 80,000 SNPs that differ from the sorghum reference genome sequence (Paterson et al., 2009). These SNPs serve as a powerful filter for removing background SNPs from BSA. For example, during the mapping of both *bm40-1* and *bm40-2*, huge number of mutations (13,419 in *bm40-1* and 11,677 in *bm40-2*) mutations were identified in the bulked *bm40-1* and *bm401-2* populations. After removing background mutations by subtracting the 80,000 SNPs annotated from the sequenced mutant lines, the numbers of raw mutations were greatly reduced (Figure 3A).

Another challenge of BSA is identification of the causal mutation from closely linked mutations, but again, this hurdle can be overcome by sequencing two or more allelic mutants. In our study, we identified 5 linked homozygous mutations in *bm40-1* and 13 in *bm40-2*. Comparison of these two populations revealed that only one gene, Sobic.001G228100, had homozygous mutations in both bulked F_2_ sequences. Furthermore, the mutation site in *bm40-1* bulked F_2_ was a missense mutation in the conserved GDSL domain, whereas the mutation in *bm40-2* bulked F2 altered the splice junction from the donor side. It is less likely for two allelic mutants with same phenotypes to have mutations in different genes. Thus, this gene is most likely the candidate for *Bm40*. Based on the average number of deleterious mutations of 147 per line in the sequenced mutant library (Jiao et al., 2016), the gene mutation rate per line per generation would about 0.004 (147/34,000) by chance. For two mutations occurred randomly in one gene would be 1.6 × 10^−5^, a nearly impossible event. Similar approach has been reported in identifying the causal mutations that affect germination of lettuce seeds under high temperatures (Huo et al., 2016). Because of the advantage of sequencing two independent alleles by BSA in overcoming the limitations of MutMap, This method should be named allelic MutMap. This method is an efficient and cost-effective approach to identifying causal gene mutations when an allelic mutant series is available. Based on current sequencing prices, the cost to sequence two bulked samples to 10 Gb data or ~15x coverage is <$500 in sorghum.

For a large number of mutants with similar phenotypes, such as the sorghum *bm* mutants, it is impractical to perform pairwise allelic test. It may be possible to sequence many bulked F_2_ populations first without prior knowledge of allelic relationship. If two or more bulked F2 population harbor unique mutations on a common gene, complementation tests can then be performed only among mutants with the same mutated gene to confirm that they are allelic. Reciprocal crosses and cosegregation analysis maybe required to further confirm the identification of the causal gene. In this manner, the time and expense required for complementation testing among large numbers of mutants could be reduced substantially. In light of the continuing decrease in sequencing costs and increase in data output and quality, it will soon be feasible to use this approach to sequence large numbers of bulked F_2_ populations. It is likely that some bulked F_2_ populations possess several linked gene mutations but no common mutated genes are found from other bulked F_2_. In these cases, complementation of the mutant phenotype with the wild type gene through transformation will be needed to confirm the identification of the causal mutation.

The importance of epicuticular wax to the drought tolerance in sorghum has long been recognized. Many *bm* mutants have been registered and genetically tested (Weibel, 1986a,b; Peters et al., 2009). A total of 38 *bm* loci were identified through genetic complementation (Peters et al., 2009), and a *bm* locus from a natural mutation was recently mapped (Uttam et al., 2017), bringing the total number of *bm* loci to 39. From the pedigreed mutant library, we identified 107 independent *bm* mutants (Jiao et al., 2016). Despite the large number of sorghum *bm* mutants collected thus far, few causal genes have been identified. Based on the *Arabidopsis* gene networks involved in EW accumulation (Kunst and Samuels, 2003; Bernard and Joubès, 2013; Fich et al., 2016), we searched the 256 sequenced core mutant lines and identified seven sorghum homologs in which mutations cause *bm* phenotypes (Jiao et al., 2016). Due to the massive accumulation of EW in sorghum, it is reasonable to speculate that sorghum employs multiple genes to regulate wax accumulation in its aerial surface. Identification of these genes and elucidation of their regulation may facilitate manipulation of surface wax accumulation in other crops with the goal of increasing their drought tolerance. To identify the genes involved in EW accumulation in sorghum, we established an efficient forward genetic method to identify the causal mutations defined by the vast collection of sorghum *bm* mutants, which can potentially identify all causal mutations of sorghum *bm* mutants in the near future.

Another GDSL-like lipase gene, Sobic.001G269200, was implicated in a *bm* QTL mapped from a natural accession RS647 (Uttam et al., 2017). However, because Sobic.001G269200 is more than 13 Mb from the *bm40* on chromosome 1 and belongs to a different clade (Figure 4A), these two GDSL lipases may represent two separate genes. To determine whether this gene is also involved in EW accumulation, we searched the sequenced mutant database (Jiao et al., 2016). We identified 10 mutations in the gene, including one stop-gain mutation in the sequenced mutant line ARS192. However, none of these 10 lines segregated for the *bm* phenotype (Supplementary Table 1). We also identified a heterozygous mutation in *Bm40*, Sobic.001G228100, in ARS29. This line was shown to segregate for the *bm* phenotype as a single recessive gene mutation (Supplementary Table 1). It is unclear if the *bm* QTL mapped from RS647 is related to *Bm40*. Nevertheless, *Bm40* is likely to be a functional orthology of the tomato *CD1* based on phenotypic similarity and protein sequence identity, but further evidence, such as enzyme assays or lipidomic profiling, is required to firmly establish whether *Bm40* is indeed a cutin synthase. The allelic mutants and identification of the causal gene provide critical materials for such future studies.

## Conclusion

Using allelic MutMap, we showed that the causal gene in *bm40* mutants encodes a GDSL-like lipase, similar to tomato cutin synthase 1. The identity of the *Bm40* gene is supported by two independent allelic mutations and a nonsynonymous mutation from a sequenced mutant library. Our results demonstrate that the allelic MutMap is an efficient and cost-effective approach for identifying causal gene mutations. This approach could be used to identify causal mutations for other phenotypes for which allelic mutants are available. Identification of the causal gene for the *bm* phenotype provides critical insight into the accumulation of massive amounts of EW on the aerial surface of sorghum plant and the superior drought tolerance of this crop.

## Disclaimer

Mention of trade names or commercial products in this article is solely for the purpose of providing specific information and does not imply recommendation or endorsement by the U.S. Department of Agriculture. USDA is an equal opportunity provider and employer.

## Author contributions

ZX and DW: Conceived the idea of this work; ZX: Isolated the mutants; ZX, GB, NG, VA-M, JC, and JB: Conducted the phenotype analysis; YJ: Analyzed the genomic data for the identification of the gene; YJ and ZX: Drafted the manuscript. All authors participated in the revision and agree with the final manuscript.

### Conflict of interest statement

The authors declare that the research was conducted in the absence of any commercial or financial relationships that could be construed as a potential conflict of interest.
